# Stroke hospitalization trends of the working-aged in Finland

**DOI:** 10.1371/journal.pone.0201633

**Published:** 2018-08-01

**Authors:** Jussi O. T. Sipilä, Jussi P. Posti, Jori O. Ruuskanen, Päivi Rautava, Ville Kytö

**Affiliations:** 1 Department of Neurology, North Karelia Central Hospital, Siun Sote, Joensuu, Finland; 2 Department of Neurology, Division of Clinical Neurosciences, Turku University Hospital, Turku, Finland; 3 Department of Neurology, University of Turku, Turku, Finland; 4 Turku Brain Injury Centre, Turku University Hospital, Turku, Finland; 5 Department of Neurosurgery, Division of Clinical Neurosciences, Turku University Hospital, Turku, Finland; 6 Department of Public Health, University of Turku, Turku, Finland; 7 Turku Clinical Research Centre, Turku University Hospital, Turku, Finland; 8 Heart Center, Turku University Hospital, Turku, Finland; 9 Research Center of Applied and Preventive Cardiovascular Medicine, University of Turku, Turku, Finland; University of L’Aquila, ITALY

## Abstract

**Background:**

The age-standardized incidence of stroke has decreased globally but, for reasons unknown, conflicting results have been observed regarding trend in incidence of major stroke subtypes in young adults. We studied these trends among people of working age in a population-based setting in Finland, where cardiovascular risk factor profiles have developed favorably.

**Methods:**

All hospitalizations for stroke in 2004–2005 and 2013–2014 for persons 18–64 years of age were identified from a national register. The search included all hospitals that provide acute stroke care on mainland Finland.

**Results:**

Hospitalizations for both intracerebral hemorrhage (ICH; -15.2%; p = 0.0008) and subarachnoid hemorrhage (SAH; -26.5%; p<0.0001) decreased overall and for both sexes separately. Concerning IS, hospitalizations decreased only for men (-6.3%; p = 0.0190) but not for women or overall. However, there was an increase in IS hospitalizations in men 35–44 years of age (+37.5%; p = 0.0019). The length of stay (LOS) of IS patients declined in nearly all subgroups (overall -20.8%, p<0.0001) whereas no change in LOS was observed for patients with ICH or SAH. In-hospital mortality decreased in patients with IS (-42.8%; p = 0.0092) but remained unchanged in patients with ICH or SAH.

**Conclusions:**

Stroke hospitalizations of young people declined in Finland, except for men 35–44 years of age for whom IS hospitalizations increased. Declining LOS and in-hospital mortality of IS patients suggests admission of less severe cases, improved care or both.

## Introduction

Cardiovascular diseases are a major cause of death and disability worldwide, but the epidemiology is changing with apparent geographical differences [1]. Although the age-standardized incidence is decreasing, the overall burden inflicted by stroke is increasing in high-income countries [2]. Moreover, the incidence of ischemic stroke (IS) is increasing in young people but it is unclear why [3]. Recent data shows that the rate of IS hospitalizations of young adults is increasing in the United States and the prevalence of traditional stroke risk factors is increasing among this cohort [4]. Interestingly, despite the similarity of their risk factor profiles compared to IS, hospitalization rates for intracerebral hemorrhage (ICH), and subarachnoid hemorrhage (SAH) remained stable [4]. On the other hand, recent prospective data from Israel shows declining IS hospitalization rates despite increasing prevalence of hypertension, dyslipidemia, diabetes mellitus, and atrial fibrillation among these patients. The declining rate was also evident in patients <55 and patients 55–64 years of age [5].

Cardiovascular risk factor levels of the Finnish population have developed favorably over the recent decades, and this has been observed also in young adults [6,7]. Indeed, the incidence of myocardial infarction has declined in Finland [8,9]. The rate of hospitalizations for SAH has declined and that for ICH has remained stable [10]. However, the rate of IS hospitalizations has increased [10]. Furthermore, from 1999 to 2008 the incidence of first-ever IS increased in persons 25–44 of age but declined in persons 55 years and older [11].

## Aims

To investigate trends of stroke hospitalizations of the working-age population in Finland over a decade.

## Methods

All discharges from neurological, neurosurgical and intensive care units with IS (ICD-10 code I63.XX), ICH (I61.X) or SAH (I60.0-I60.6) as the primary diagnosis between January 1, 2004-December 31, 2005 and January 1, 2013-December 31, 2014 were identified from the Care Register for Health Care (CRHC), a mandatory database for all public health care hospital discharges in Finland. The study period was modelled on the recent study on the subject from the United States [4] to enhance comparability. Patients with a primary diagnosis of ICH and SAH with secondary diagnoses of arteriovenous malformations (Q28.X) and traumatic brain injury (S06.X) were excluded. All 22 university and central hospitals, which are the only hospitals on mainland Finland to provide acute stroke care, were included in the search. Hospital transfers related to a particular episode of a hospitalization were combined as one admission. Only patients 18–64 years of age were included. Discharge destination was available as home, care facility (skilled nursing units/rehabilitation facilities/long-term care facility) or deceased. General population demographic data was obtained from Statistics Finland. The background population at risk consisted of 13,165,993 person-years. Population-adjusted hospitalization rates for pre-determined age cohorts were modelled after the recent study from the United States [4]. The study was reviewed and approved by the Hospital District of Southwest Finland (T236/2014) and the National Institute for Health and Welfare of Finland (permissions no: THL/143/5.05.00/2015 and THL/1349/5.05.00/2015). Since all data was anonymized by the provider (THL) before delivery to the researchers and the study involved no contact with the patients the Finnish law does not require a handling by an ethics committee.

Relative changes were calculated as (value in 2013–2014 / value in 2004–2005) / value in 2003–2004. Hospitalization rates were analyzed using Poisson regression modelling with logarithm of population risk as an off-set parameter. Cox regression was used for analysis of in-hospital mortality. Wilcoxon-Mann-Whitney test was used for analysis of length of hospital stay. Proportion of ischemic stroke patients who received intravenous thrombolysis were analyzed with Chi-squared test. Statistical significance was inferred at P-value <0.05. All analyses were conducted using SAS System for Windows, version 9.4 (SAS Institute Inc., Cary, NC, USA).

## Results

We identified 10976 stroke hospitalizations (63.3% male), of which 7680 were due to IS, 1664 due to ICH and 1632 due to SAH.

### Ischemic stroke

Overall hospitalizations for IS remained unchanged (Table 1). In women, no age group showed change, but in men a 6.3% overall decrease was observed. Furthermore, there was an increase in men 35–44 years of age and for both sexes combined in that age group. There was a decrease of hospitalizations in men aged 55–64 years and for the sexes combined in this age group.

**Table 1:** Stroke frequency and yearly hospitalization rate / 100’000 persons.

	Ischemic stroke						Intracerebral hemorrhage						Subarachnoidal hemorrhage					
	2004–2005		2013–2014		Relative Change (%)	P	2004–2005		2013–2014		Relative Change (%)	P	2004–2005		2013–2014		Relative Change (%)	P
	N	Rate	N	Rate	Rate		N	Rate	N	Rate	Rate		N	Rate	N	Rate	Rate	
**Total 18–64**	3921	59.6	3759	57.1	-4.3	0.0550	900	13.7	764	11.6	-15.2	0.0008 (*)	940	14.3	692	10.5	-26.5	< .0001 (*)
**Male**	2655	79.9	2495	74.9	-6.3	0.0190 (*)	581	17.5	497	15.0	-14.7	0.0091 (*)	437	13.2	288	8.6	-34.3	< .0001 (*)
**Female**	1266	38.9	1264	38.8	-0.2	0.97	319	9.8	267	8.2	-16.3	0.0320 (*)	503	15.5	404	12.4	-19.7	0.0010 (*)
**Age 18–34**	118	5.4	154	6.6	23.1	0.089	33	1.5	41	1.8	17.2	0.50	63	2.9	36	1.6	-46.1	0.0031 (*)
**Male**	59	5.3	84	7.1	34.0	0.085	20	1.8	29	2.4	36.5	0.28	30	2.7	17	1.4	-46.7	0.0384 (*)
**Female**	59	5.5	70	6.2	12.2	0.51	13	1.2	12	1.1	-12.7	0.73	33	3.1	19	1.7	-45.6	0.0347 (*)
**Age 35–44**	283	19.5	338	25.8	32.8	0.0004 (*)	89	6.1	65	5.0	-18.8	0.20	188	12.9	119	9.1	-29.6	0.0027 (*)
**Male**	171	23.1	213	31.8	37.5	0.0019 (*)	57	7.7	44	6.6	-14.8	0.42	89	12.0	46	6.9	-43.0	0.0020 (*)
**Female**	112	15.7	125	19.6	25.2	0.085	32	4.5	21	3.3	-26.4	0.27	99	13.8	73	11.4	-17.3	0.22
**Age 45–54**	1003	64.9	937	63.7	-1.8	0.68	274	17.7	199	13.5	-23.7	0.0037 (*)	354	22.9	247	16.8	-26.7	0.0002 (*)
**Male**	684	88.1	639	86.3	-2.0	0.72	174	22.4	123	16.6	-25.8	0.0113 (*)	179	23.0	110	14.9	-35.5	0.0003 (*)
**Female**	319	41.6	298	40.8	-1.7	0.83	100	13.0	76	10.4	-20.0	0.14	175	22.8	137	18.8	-17.6	0.0889
**Age 55–64**	2517	180.7	2330	157.0	-13.7	< .0001 (*)	504	36.2	459	30.7	-15.1	0.0114 (*)	335	24.0	290	19.4	-13.4	0.0076 (*)
**Male**	1741	253.0	1559	212.0	-16.2	< .0001 (*)	330	48.0	301	40.9	-14.6	0.0469 (*)	139	20.2	115	15.6	-17.3	0.0423 (*)
**Female**	776	110.1	771	101.7	-7.6	0.11	174	24.7	158	20.8	-15.6	0.12	196	27.8	175	23.1	-17.0	0.073

The relative change is presented for the hospitalization rate. P is presented according to Poisson regression.

*, p<0.05

The overall in-hospital mortality of IS patients decreased with a particular decline in the age group 35–44 years of age (Table 2). The length of stay of IS patients declined in practically all subgroups (Table 3). The proportion of IS patients discharged alive but transferred to a care facility increased overall and this was driven by patients aged 55–64 years Table 4). The proportion of patients receiving iv thrombolysis nearly doubled to 5.3% (Table 5).

**Table 2:** In-hospital mortality.

	Ischemic stroke				Intracerebral hemorrhage				Subarachnoidal hemorrhage			
	2004–2005 (%)	2013–2014 (%)	Relative change (%)	P	2004–2005 (%)	2013–2014 (%)	Relative change (%)	P	2004–2005 (%)	2013–2014 (%)	Relative change (%)	P
Total 18–64	2.8	1.6	-42.8	0.0092 (*)	13.6	13.7	1.3	0.84	9.9	9.0	-9.4	0.72
Male	3.2	1.8	-41.8	0.0373 (*)	12.7	14.9	16.9	0.32	9.6	9.7	1.1	0.82
Female	2.1	1.2	-44.1	0.11	15.1	11.6	-22.9	0.28	10.1	8.4	-17.0	0.48
Age 18–34	2.5	0.7	-74.4	0.25	9.1	12.2	34.2	0.74	4.8	2.8	-41.6	0.63
Male	1.7	1.2	-29.6	0.89	5.0	10.3	106.8	0.77	6.7	5.9	-11.8	0.98
Female	3.4	0.0	-100	1.00	15.4	16.7	8.4	0.83	3.0	0	-100	1.00
Age 35–44	3,9	0.3	-92.3	0.0171 (*)	9.4	23.1	156.7	0.0304 (*)	9.6	4.2	-56.1	0.14
Male	3.5	0.5	-86.6	0.095	8.8	27.3	210.9	0.0297 (*)	7.9	4.4	-44.7	0.41
Female	4.5	0.0	-100	0.99	9.4	14.3	52.3	0.60	11.1	4.1	-63.0	0.20
Age 45–54	2.2	1.3	-41.6	0.29	14.2	9.1	-36.4	0.093	9.3	7.7	-17.5	0.58
Male	2.8	1.4	-49.3	0.17	11.5	9.8	-15.1	0.60	10.1	8.2	-18.7	0.74
Female	0.9	1.0	7.4	0.78	19.0	7.9	-58.5	0.051	8.6	7.3	-14.8	0.65
Age 55–64	3.0	2.0	-32.2	0.21	14.3	14.6	2.2	0.77	11.6	12.8	9.6	0.55
Male	3.3	2.3	-32.4	0.31	14.6	15.6	7.3	0.65	10.8	13.0	28.9	0.38
Female	2.2	1.6	-28.8	0.50	13.8	12.7	-8.2	0.90	12.2	12.0	-2.0	0.94

P is presented according to Cox regression.

*, p<0.05

**Table 3:** Length of stay (LOS).

	Ischemic stroke						Intracerebral hemorrhage						Subarachnoidal hemorrhage					
	2004–2005		2013–2014		Relative Change (%)	P	2004–2005		2013–2014		Relative Change (%)	P	2004–2005		2013–2014		Relative Change (%)	P
	mean	SD	mean	SD			mean	SD	mean	SD			mean	SD	mean	SD		
**Total 18–64**	10.2	11.2	8.0	8.9	-20.8	< .0001 (*)	13.9	17.1	11.8	12.7	-15.4	0.34	13.5	14.0	13.2	12.9	-2.4	0.65
**Male**	10.2	10.9	8.1	8.9	-20.2	< .0001 (*)	14.1	17.8	11.2	11.2	-20.1	0.25	13.4	14.2	12.0	10.3	-10.4	0.63
**Female**	10.0	11.8	7.8	9.1	-21.9	< .0001 (*)	13.6	15.8	12.8	15.0	-6.4	0.96	13.7	13.8	14.1	14.4	2.9	0.83
**Age 18–34**	11.7	14.1	9.9	9.7	-15.5	0.25	13.6	21.3	12.5	15.9	-8.0	0.13	13.0	11.9	13.3	11.5	2.6	0.81
**Male**	11.3	9.2	9.6	10.8	-15.1	0.0151 (*)	10.1	18.2	11.7	9.9	16.7	0.054	12.2	10.1	9.8	10.4	-19.7	0.36
**Female**	12.0	17.8	10.2	8.2	-15.5	0.35	19.0	25.1	14.3	25.7	-24.6	1.00	13.8	13.4	16.5	11.1	20.1	0.24
**Age 35–44**	11.0	14.8	7.9	7.5	-27.9	0.0061 (*)	14.0	17.5	12.4	14.2	-11.0	0.84	13.6	13.8	12.7	11.1	-6.3	0.84
**Male**	10.8	13.1	7.8	7.0	-28.1	0.0086 (*)	15.9	20.6	12.2	12.2	-23.0	0.72	13.7	12.3	14.1	10.8	3.1	0.66
**Female**	11.4	17.0	8.2	8.2	-27.4	0.30	10.5	9.3	12.8	18.0	21.6	0.96	13.5	15.0	11.8	11.3	-12.1	0.62
**Age 45–54**	9.7	10.3	7.8	9.0	-19.8	< .0001 (*)	15.4	19.6	13.3	13.6	-13.5	0.98	13.4	14.3	13.1	13.2	-2.1	0.70
**Male**	9.6	9.9	8.3	9.2	-13.8	< .0001 (*)	16.2	22.1	12.6	11.4	-22.0	0.99	13.5	14.6	11.6	10.1	-14.0	0.56
**Female**	10	11.2	6.8	8.7	-32.2	< .0001 (*)	14.0	14.2	14.4	16.5	2.8	0.89	13.3	14.0	14.3	15.2	7.9	1.00
**Age 55–64**	10.2	10.9	8.0	9.0	-21.0	< .0001 (*)	13.1	15.2	11.0	11.6	-16.6	0.18	13.8	14.2	13.5	13.5	-2.1	0.69
**Male**	10.4	11.1	8.1	8.9	-22.2	< .0001 (*)	12.9	14.4	10.5	11.1	-18.7	0.11	13.2	15.6	11.8	10.3	-10.8	0.99
**Female**	9.7	10.5	8.0	9.4	-18.0	< .0001 (*)	13.4	16.7	11.8	12.7	-12.9	0.90	14.2	13.1	14.6	15.1	3.0	0.65

P is presented according to Wilcoxon-Mann-Whitney test (Two-sided).

*, p<0.05; SD, standard deviation

**Table 4:** The proportion of patients who were transferred to skilled nursing units/rehabilitation facilities/long-term care facilities from the hospital.

	Ischemic stroke				Intracerebral hemorrhage				Subarachnoidal hemorrhage			
	2004–2005 (%)	2013–2014 (%)	Relative change (%)	P	2004–2005 (%)	2013–2014 (%)	Relative change (%)	P	2004–2005 (%)	2013–2014 (%)	Relative change (%)	P
**Total 18–64**	43.4	48.5	11.9	< .0001 (*)	61.8	76.9	24.4	< .0001 (*)	51.8	66.8	28.9	< .0001 (*)
**Male**	44.9	50.7	13.0	< .0001 (*)	63.9	79.4	24.3	< .0001 (*)	51.7	65.4	26.6	0.0005 (*)
**Female**	40.3	44.2	9.8	0.0477 (*)	57.9	72.5	25.1	0.0006 (*)	52.0	67.8	30.5	< .0001 (*)
**Age 18–34**	42.6	43.1	1.2	0.93	50.0	77.8	55.6	0.0184 (*)	55.0	40.0	-27.3	0.16
**Male**	43.1	47.0	9.0	0.65	47.4	84.6	78.6	0.0077 (*)	53.6	62.5	16.7	0.57
**Female**	42.1	38.6	-8.4	0.69	54.6	60.0	10.0	0.80	56.3	21.1	-62.6	0.0141 (**)
**Age 35–44**	37.9	44.8	18.3	0.084	55.6	76.0	36.8	0.0183 (*)	45.9	65.8	43.4	0.0010(*)
**Male**	39.4	46.7	18.6	0.16	57.7	78.1	35.4	0.056 (*)	48.8	61.4	25.8	0.18
**Female**	35.5	41.6	17.2	0.34	51.7	72.2	39.6	0.16	43.2	68.6	58.8	0.0015(*)
**Age 45–54**	40.3	42.0	4.2	0.46	63.4	76.8	21.1	0.0033 (*)	52.0	61.8	18.9	0.0224(*)
**Male**	41.2	44.8	8.6	0.20	65.0	80.2	23.5	0.0068(*)	50.3	57.4	14.2	0.26
**Female**	38.3	35.9	-6.2	0.55	60.5	71.4	18.1	0.16	53.8	65.4	21.6	0.0472 (*)
**Age 55–64**	45.3	52.0	15.0	< .0001 (*)	63.0	77.0	22.4	< .0001 (*)	54.4	75.5	38.8	< .0001 (*)
**Male**	46.9	53.9	14.9	< .0001 (*)	65.6	78.7	20.0	0.0007 (*)	54.8	75.8	38.1	0.0012 (*)
**Female**	41.6	48.4	16.1	0.0085 (*)	58.0	73.9	27.4	0.0045 (*)	54.1	75.3	29.3	< .0001 (*)

The patients who died while in hospital are not included in this analysis. P is presented according to Chi-squared.

*, p<0.05

**Table 5:** The proportion of patients with an ischemic stroke (IS) who received intravenous thrombolysis.

	2004–2005 (%)	2013–2014 (%)	Relative change (%)	P
**Total 18–64**	2.8	5.3	90.9	< .0001 (*)
**Male**	2.7	4.6	70.1	0.0003 (*)
**Female**	2.8	6.6	131.3	< .0001 (*)
**Age 18–44**	2.3	5.7	152.9	0.0104 (*)
**Male**	2.2	5.1	131.7	0.088
**Female**	2.2	6.7	197.8	0.0497 (*)
**Age 45–56**	2.8	5.2	85.4	< .0001 (*)
**Male**	2.8	4.6	64.9	0.0011 (*)
**Female**	2.9	6.6	12.,3	< .0001 (*)

P is presented according to Chi squared.

*, p<0.05

### Intracerebral hemorrhage

Hospitalizations for ICH decreased overall (Table 1). This was driven primarily by decrease in men 45–64 years of age, although a decrease in hospitalizations overall was observed also in women. In-hospital mortality of ICH patients changed only in men aged 35–44 years for whom mortality increased (Table 2). No change in LOS was observed (Table 3). The proportion of ICH patients discharged alive who were transferred to a care facility increased overall and this was evident in nearly all subcategories as well (Table 4).

### Subarachnoid haemorrhage

SAH hospitalizations declined in both sexes, but particularly in men for whom change was observed in all age categories. Albeit there was a decline in overall female SAH hospitalizations, no change among women over 35 years of age was observed. No change in LOS or in-hospital mortality was observed in any SAH patient group (Tables 1–3). The proportion of SAH patients discharged alive but transferred to a care facility increased overall. However, the proportion of transferred patients among the youngest women declined while no change was observed among men of same age (Table 4).

## Discussion

This nationwide study showed that stroke hospitalization frequencies of working-aged persons has declined in Finland. However, for ischemic stroke overall this was observed only in men. Furthermore, IS hospitalization subgroup analyses showed opposite trends for both men and sexes combined in age groups of 35–44 and 55–64 years. Overall, our results are congruent with the previously reported decline in age-adjusted incidence rate of stroke in high-income countries [12–14].

Our findings probably stem from favorable development in cardiovascular risk factors of the Finnish population [6,7,15] The incidence of SAH has decreased in Finland [15] This has been primarily attributed to a decline in smoking [16–18]. Our results indicate a decline in SAH hospitalizations in all studied male age groups, but for women the decline was only observed in the youngest age group. This may also be linked to smoking trends in Finland, as during the years 2004–2014 the proportion of daily male smokers aged 15–64 years decreased by 36% while the decrease was 25% among women of the same age [19]. This is all the more pertinent as it has been recently reported that female gender might not be an independent risk factor for SAH, but smoking was suggested to have a stronger dose-dependent risk association in women than in men [16].

The overall hospitalization frequency for ICH in Finland has not changed over 2004–2014 [11]. Considering the current result of a declining trend of ICH hospitalizations in the working-aged it seems that ICH occurrence in Finland is shifting to ever older age groups, contingently due to the improved cardiovascular risk profile in adults [6] and increasing proportion of older people in the Finnish population.

It is unknown why the incidence of young-onset IS has increased [3]. Interestingly, prospective registry data from 1983–1997 showed a declining trend of IS incidence in two regions of Finland for both men and women aged 25–54 years [20]. However, from 1999 to 2008 there was an increase of first-ever IS rates in people 25–44 years of age and declining rates in those over 55 years of age [11], in line with our results. It appears plausible that changes in diagnostics and case definitions that have previously been suggested [21] as reasons for the increase might be behind this. The diagnostic process of IS is fraught with more difficulty and uncertainty than that of hemorrhagic stroke. After the turn of the millennium, the increased availability of MRI and perfusion CT imaging means more cases of minor or transient symptoms can now be recognized to be of ischemic origin. This might be of particular relevance in younger patients, in whom IS is more easily missed initially [22]. Also, the current, tissue-based definition of a transient ischemic attack (TIA), may have resulted in more cases of transient symptoms to be classified as infarcts as compared to the previous, time-dependent definition [21]. This might be particularly relevant in young patients, who recover faster compared to older people [23,24]. MRI imaging has been reported to have no impact on IS incidence [25], but patient age was not evaluated as a contributing factor. This is important because young IS patients are more often imaged with MRI [26,27].

We consider our results to be in keeping with these explanations. Considering the many similarities in risk factor profiles of IS and hemorrhagic stroke, it seems logical that their true incidence would decline in a roughly similar fashion when population risk factor profiles have improved. The rise in IS hospitalizations of men aged 35–44 years is perfectly explicable with the aforementioned changes in diagnostics and case definitions that probably have most impact in this age group, as the absolute numbers of cases in younger age groups make it improbable that statistically significant changes would occur and the increasing importance of traditional risk factors in older age groups make it less likely that cases would have been missed previously.

Furthermore, the decline in LOS and the nearly halved in-hospital mortality suggest that an increase in hospitalization of less serious cases of working-aged IS patients has occurred. This was most apparent in the only group where increased hospitalizations was observed, namely men aged 35–44 with IS. This is in line with a recent study from Israel, which reported nationwide prospective data and indicated that from 2004 to 2013 stroke severity in all hospitalized IS patients decreased and the trend was also clearly evident in the working-aged [5]. That study reported declining stroke hospitalization rates also for the working-aged, similarly to our results. Naturally, the decline in LOS and mortality in our data may also be partially explained by improved care reported in Finland previously [12], evident also in our study as increased, although still low, thrombolysis rates. One factor to consider in the increase in IS hospitalizations of men 35–44 years of age is illicit drug use [28]. Unfortunately, we do not have data concerning drug use at our disposal. However, this seems an unlikely explanation considering that although illicit drug use has increased in Finland, it is concentrated mainly to people under 35 years of age [29] for whom no increase in stroke hospitalizations was observed. Lastly, considering that TIA incidence has also been reported to have increased in the working-aged [30,31], it seems probable that increased awareness might also play a role. Unfortunately, we do not have solid data concerning TIA occurrence at our disposal as many of these patients are not admitted to wards but treated as outpatients. Taking all this into account, we suggest that in populations that show improving cardiovascular risk profiles, adult-onset IS is only better detected, not truly more frequent.

With no change in in-hospital mortality or LOS for SAH and ICH, it appears that the case and acute treatment profiles have not markedly changed for them during the decade. The proportion of discharges from hospital to a nursing home or similar placement may reflect a more intensive efforts in subacute phase rehabilitation, as the change was observed for all stroke subtypes.

This was a retrospective registry study and therefore there are certain weaknesses that need to be observed. The main problem is the lack of clinical data, such as NIHSS scores, medications beyond thrombolysis, and imaging data. Furthermore, there is always some uncertainty as to the accuracy of the data, which was gathered primarily for administrative purposes. Naturally, our results cannot be used as population-based total incidence figures for stroke as we had no data on cases that did not reach hospital. However, these challenges are counter-balanced by the nationwide extent of the data and the fact that the CRHC has been found to be reliable for these purposes [32].

Our results suggest that further studies investigating the impact of modern diagnostics on IS incidence are needed. Overall, these results create faith in the public health measures that have been introduced to tackle the high level of cardiovascular disease in Finland.
